# A Case of Proliferative Glomerulonephritis with Monoclonal IgG Deposits That Showed Predominantly Membranous Features

**DOI:** 10.1155/2017/1027376

**Published:** 2017-10-25

**Authors:** Homare Shimohata, Kentaro Ohgi, Hiroshi Maruyama, Yasunori Miyamoto, Mamiko Takayashu, Kouichi Hirayama, Masaki Kobayashi

**Affiliations:** Department of Nephrology, Tokyo Medical University Ibaraki Medical Center, Ibaraki, Japan

## Abstract

In 2004, the novel category of monoclonal IgG deposition disease has been proposed and termed “proliferative glomerulonephritis with monoclonal IgG deposits” (PGNMID). This disease is characterized by membranoproliferative glomerulonephritis and staining for a single light-chain isotype and gamma heavy-chain subclass. A 76-year-old male who had monoclonal gammopathy was referred to our hospital because of proteinuria. The renal biopsy showed diffuse thickening of the glomerular capillary walls with focal mesangial proliferation. On immunofluorescence study, only IgG1 among the four subclasses and lambda light chains were detected mainly in the glomerular capillary walls. From these results, we diagnosed our case as PGNMID showing predominantly membranous features. Almost all pathological findings on light microscopy of PGNMID are membranoproliferative GN or endocapillary proliferative GN, while membranous GN cases are rare. Here, we present the case of PGNMID that showed predominantly membranous features on light microscopy.

## 1. Introduction

Although AL amyloidosis and monoclonal immunoglobulin deposition disease (light-chain deposition disease and heavy-chain deposition disease, resp.) are both characterized by monoclonal immunoglobulin deposits in tissues, these diseases are distinguishable strictly by Congo red staining and characteristic appearance on electron microscopy. Furthermore, almost all fragments of light-chain deposition in AL amyloidosis are of the lambda type, whereas LCDD depositions are of the kappa type. In 2004, Nasr et al. reported the novel category of monoclonal IgG deposition disease characterized by membranoproliferative glomerulonephritis or endocapillary glomerulonephritis on light microscopic findings, staining for a single light-chain isotype and a single gamma heavy-chain subclass on immunofluorescence findings, and granular electron-dense deposits on electron microscopic findings [1]. Thereafter, they gathered 37 similar cases and termed this novel category of glomerular involvement “proliferative glomerulonephritis with monoclonal IgG deposits” (PGNMID) [2]. After their disease concept proposal, further cases were reported by other groups [3, 4].

According to Nasr et al.'s report, almost all pathological findings on light microscopy of PGNMID are membranoproliferative GN or endocapillary proliferative GN, while membranous GN cases are rare. Here, we present the case of PGNMID that showed predominantly membranous features on light microscopy.

## 2. Case Presentation

**Figure 1:**
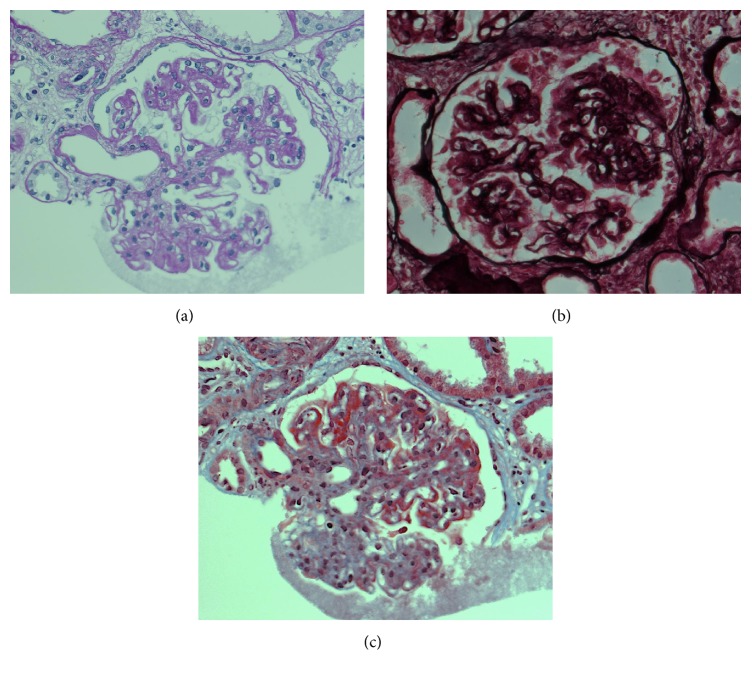
Light microscopy findings. (a) Periodic acid-Schiff stain (PAS) staining showed focal mesangial proliferation (original magnification ×400). (b) Periodic acid-methenamine silver (PAM) staining showed thickening of glomerular capillary walls (original magnification ×400). (c) Masson trichrome (MT) staining showed red staining in the subepithelial area (original magnification ×400).

**Figure 2:**
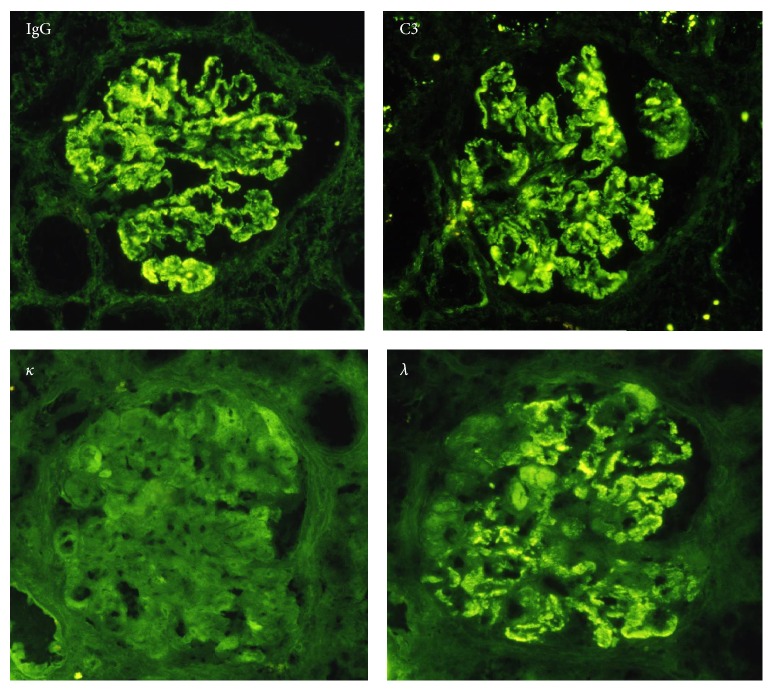
Immunofluorescence findings. IgG was strongly positive mainly in the peripheral capillary walls. C3 was also positive in peripheral pattern. In light-chain staining, kappa chain was entirely negative, but lambda chain was positive in the peripheral capillaries (original magnification ×400).

**Figure 3:**
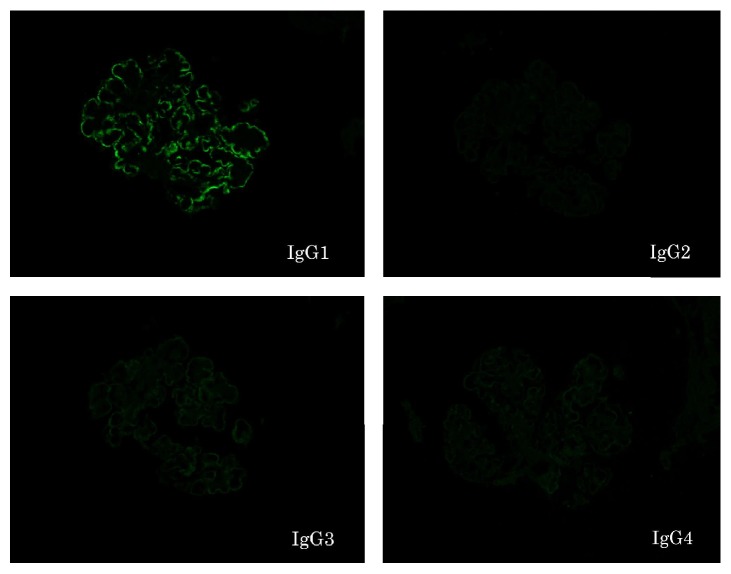
Findings of IgG subclass staining. IgG1 was positive in peripheral granular pattern. On the other hand, IgG2, IgG3, and IgG4 were all negative (original magnification ×400).

**Figure 4:**
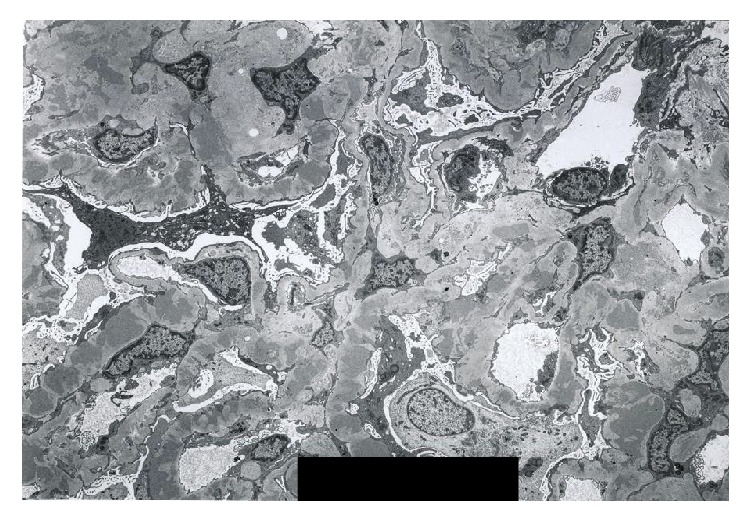
Electron microscopic finding. Electron microscopy showed huge electron-dense deposits in subepithelial, intramembranous, and mesangial lesions (original magnification ×3,000).

A 76-year-old Japanese male was referred to our hospital because of pretibial edema and proteinuria. On examination, his blood pressure was 124/90 mmHg and the pulse rate was 74 beats/min (regular sinus rhythm). Laboratory data showed serum creatinine and blood urea nitrogen of 3.6 and 46.7 mg/dL, respectively. Hemoglobin and serum albumin were 9.7 and 2.7 g/dL, respectively. C-reactive protein, alanine aminotransferase, aspartate aminotransferase, lactate dehydrogenase, blood glucose, hemoglobin A1c, and electrolytes were all normal. Serum cryoglobulin, hepatitis B virus surface antigen, and hepatitis C virus antibodies were negative. Urinalysis showed proteinuria (4.9 g/day) and hematuria (5–9 erythrocytes per high-power field) with granular casts (5–9 per high-power field) and lipid casts (5–9 per whole field). Proteinuria was in the nephrotic range. Immunoglobulin and complement levels were all within normal limits. We detected monoclonal IgG-kappa protein, not Bence-Jones protein, by serum and urine immunoelectrophoretic study. Because the bone marrow examination showed 3.3% plasma cells and the patient had no features of hematologic malignancy, he was diagnosed with monoclonal gammopathy of renal significance [5]. Renal biopsy was performed to investigate the reason for the proteinuria and elevated serum creatinine levels. Light microscopy showed diffuse thickening of the glomerular capillary walls with focal mesangial proliferation (Figures 1(a) and 1(b)), and red staining was observed in the subepithelial area by Masson's trichrome stain (Figure 1(c)). Severe tubular atrophy, interstitial fibrosis, and monocyte infiltration were observed in the tubulointerstitium. On immunofluorescence study, IgG and C3 granular deposits were detected in the peripheral capillary walls. Moreover, only IgG1 among the four subclasses (Binding-Site, Birmingham, UK) and only lambda light chains were detected in the glomerular capillary walls (Figures 2 and 3). Granular electron-dense deposits were observed in subepithelial, intramembranous, and mesangial area by electron microscopy (Figure 4). From the above pathological findings, we considered that our case was consistent with the conception of PGNMID. Thereafter, in spite of conservative therapy such as angiotensin-converting enzyme inhibitors, calcium blocker, and erythropoietin-stimulating agents, hemodialysis therapy was started because of the progression to end-stage renal disease two years after the renal biopsy.

## 3. Discussion

Glomerular disease with monoclonal immunoglobulin deposition is divided into two categories by electron microscopy, those with organized deposits and those with disorganized deposits [6]. Amyloidosis, type 1 cryoglobulinemic glomerulonephritis, and immunotactoid glomerulonephritis are included in the first group and monoclonal immunoglobulin deposition disease is included in the second group. In 2004, Nasr et al. reported a novel form of glomerular injury related to monoclonal IgG deposition and termed the disease “proliferative glomerulonephritis with monoclonal IgG deposits” [4]. Although they described typical features of light microscopy as membranoproliferative glomerulonephritis or endocapillary proliferative glomerulonephritis, various histologic patterns have been reported. However, according to their report, membranous glomerulonephritis is a rare pathological finding in PGNMID, like mesangial proliferative glomerulonephritis. In the present report, we report a case of membranous glomerulonephritis associated with focal mesangial proliferation with monoclonal IgG deposits.

 Komatsuda et al. reported a case of immunoglobulin deposition disease with a membranous pattern. Their case showed IgG lambda bands in serum electrophoresis and IgG1-lambda immunofluorescence staining in glomeruli. Although the pathological features and underling disease were similar to our case, their case had no mesangial hypercellularity whereas our case showed a serum light-chain isotype that differed from the glomerular immune deposits. The reason for this discrepancy is not obvious, but there have been some previous reports of glomerulonephritis with monoclonal IgG deposits without detection of monoclonal proteins in serum or LCDD without the deposition of light chains in the glomerulus [7, 8]. Furthermore, Nasr et al. demonstrated that thirty percent of PGNMID patients had a detectable circulating monoclonal protein with the same light-chain isotype as the glomerular deposits [9]. Therefore, discordance between the light-chain isotypes of serum and glomerular deposits is not a rare situation.

Komatsuda et al. also reported monoclonal immunoglobulin deposition disease associated with membranous features [10]. They described the light microscopic findings of their cases as thickening of the glomerular capillary walls and spike formation without proliferative lesions and immunofluorescence staining of glomerular deposits that were all of IgG-kappa type. Furthermore, steroid therapy was very effective in their cases and renal function was preserved in all patients. On the other hand, Nasr et al. summarized clinical outcomes of 32 patients who had PGNMID, of whom 21.9% progressed to end-stage renal disease [4]. At the time of renal biopsy, our patient showed the elevation of serum creatinine, and he progressed to end-stage renal disease within two years after renal biopsy. This poor clinical outcome was similar to those of Nasr et al.'s report. Light microscopy of our case revealed membranous glomerulonephritis with focal mesangial proliferation and lambda-type light-chain deposits. These findings are more similar to the pathological features of PGNMID. From these above considerations, we diagnosed our case as PGNMID with a predominantly membranous glomerulonephritis character. It is unclear whether monoclonal immunoglobulin deposition disease associated with membranous features reported by de Seigneux et al. and Komatsuda et al. is a distinct disease concept from PGNMID. Further accumulation of cases of monoclonal immunoglobulin deposition disease associated with membranous features is needed to confirm whether these pathological features belong to PGNMID.

Here, we present the case of PGNMID that showed predominantly membranous features on light microscopy. Whenever membranous glomerulonephritis with monoclonal gammopathy is diagnosed, immunoglobulin staining for IgG subclass and immunoglobulin light chains should be conducted to clarify the immunoglobulin deposit disease.
